# Thickness-independent scalable high-performance Li-S batteries with high areal sulfur loading via electron-enriched carbon framework

**DOI:** 10.1038/s41467-021-24873-4

**Published:** 2021-07-26

**Authors:** Nana Wang, Xiao Zhang, Zhengyu Ju, Xingwen Yu, Yunxiao Wang, Yi Du, Zhongchao Bai, Shixue Dou, Guihua Yu

**Affiliations:** 1 Institute for Superconducting and Electronic Materials, University of Wollongong, Innovation Campus, Squires Way, Wollongong, NSW Australia; 2 Materials Science and Engineering Program and Department of Mechanical Engineering, The University of Texas at Austin, Austin, TX USA

**Keywords:** Energy storage, Materials science

## Abstract

Increasing the energy density of lithium-sulfur batteries necessitates the maximization of their areal capacity, calling for thick electrodes with high sulfur loading and content. However, traditional thick electrodes often lead to sluggish ion transfer kinetics as well as decreased electronic conductivity and mechanical stability, leading to their thickness-dependent electrochemical performance. Here, free-standing and low-tortuosity N, O co-doped wood-like carbon frameworks decorated with carbon nanotubes forest (WLC-CNTs) are synthesized and used as host for enabling scalable high-performance Li-sulfur batteries. EIS-symmetric cell examinations demonstrate that the ionic resistance and charge-transfer resistance per unit electro-active surface area of S@WLC-CNTs do not change with the variation of thickness, allowing the thickness-independent electrochemical performance of Li-S batteries. With a thickness of up to 1200 µm and sulfur loading of 52.4 mg cm^−2^, the electrode displays a capacity of 692 mAh g^−1^ after 100 cycles at 0.1 C with a low E/S ratio of 6. Moreover, the WLC-CNTs framework can also be used as a host for lithium to suppress dendrite growth. With these specific lithiophilic and sulfiphilic features, Li-S full cells were assembled and exhibited long cycling stability.

## Abstract (web summary)

Improving the energy density of lithium-sulfur batteries is necessary for their practical application. Here, the authors report free-standing and low-tortuosity carbon frameworks as host for sulfur and lithium, enabling scalable thickness independent electrochemical performance.

## Introduction

Great efforts are needed to enable lithium-ion batteries (LIBs) with superior energy storage capability to meet the ever-growing demand for transportation and stationary electric grids^1^. To achieve higher energy density, electrode materials with intrinsic high specific capacity must be explored, and their areal capacity must be maximized simultaneously. Sulfur cathodes, based on the conversion-reaction electrochemistry in lithium−sulfur (Li−S) batteries, have become attractive in the last few decades due to their high theoretical capacity of 1675 mAh g^−1^ and high theoretical specific energy density of 2567 Wh kg^−1^, as well as their low cost and wide availability^2–4^. The application of Li−S batteries faces some key inherent problems; however, including the insulating nature of both sulfur and its discharge products, the shuttle effect of soluble long-chain lithium polysulfides ((LiPSs) in the organic electrolyte, and serious volume expansion (~78%) of the sulfur during the discharge process, which seriously limit their cycle life and rate performance^2–4^. Various strategies have been adopted to address these issues, for instance, by encapsulating sulfur in conductive carbon/polymer composites or oxides/sulfides to chemically/physically immobilize the sulfur/LiPSs and to boost their electrochemical redox kinetics^5^. Nonetheless, the high capacity and long cycle life of Li−S batteries in most studies were obtained with relatively insufficient sulfur content and/or low sulfur loading (<4.0 mg cm^−2^), as well as excess electrolyte (>11 μL mg^−1^), which are far from the conditions necessary for their practical application^6,7^.

Therefore, it is essential to escalate the areal capacity of cathodes by increasing sulfur loading with higher electrode thickness. Nevertheless, there are some critical issues in the traditional thick electrode approach. First, the conventional thick electrode fabricated by slurry-casting on metallic current collectors suffers from fracturing and delamination due to high shrinkage stresses of the slurry during the drying processes, causing mechanical instability and poor adhesion between the active material and the current collector above the critical cracking thickness^8,9^. Second, the electrode thickness leads to sluggish charge (ion and electron) transfer kinetics and a proportional increase in the ion/electron transport distance. Much worse, the binder, as an electrically insulating component, blocks the diffusion paths of electrons and decreases the electrical conductivity of the electrode, especially for thick electrodes^10,11^. Only limited studies have focused on developing high-loading sulfur cathodes^12–15^, such as layer-by-layer sulfur cathodes with the high sulfur loading of 11.4 mg cm^−2,^^12^, and N, S co-doped graphene sponge with sulfur loading of 8.5 mg cm^−2,^^13^.

Here, a N, O co-doped three-dimensional wood-like carbon framework decorated with carbon nanotubes (CNTs) forest (denoted as WLC-CNTs) has been synthesized via the ice-templating method and followed by a tip growth of carbon nanotube forest^16,17^. Because of the high conductivity and robust wood-like features, this material can be used as a host for higher sulfur loading (S@WLC-CNTs) without any current collector, conductive additive, or binders. Such unique S@WLC-CNTs cathodes with low-tortuosity microchannels can decrease the charge (ion and electron) diffusion paths, allow the electrolyte to shuttle freely inside the cathode, and accommodate the volume changes of sulfur. Besides, the inside carbon nanotubes forest can effectively trap soluble LiPSs and catalyze their redox kinetics within the electrode through their nonpolar-polar interaction and electron-rich characteristic^18^. Most importantly, it is a thickness-independent electrode, so its areal sulfur loading can be easily scaled up by increasing thickness without sacrificing its electrochemical performance. On increasing its thickness to 1200 μm with a high areal sulfur loading of 52.4 mg cm^−2^, after 100 cycles, a reversible capacity of 692 mAh g^−1^ could still be maintained. Interestingly, WLC-CNTs can also be used as a host for lithium anode to suppress its dendrite growth (Li/WLC-CNTs), exhibiting quite small overpotential and high Coulombic efficiency (CE). The assembled Li−S full cells displayed long cycling stability with low-capacity decay per cycle (0.057%). This strategy has allowed us to produce an extremely thick electrode with extremely high sulfur loading and pave a new way for designing the high gravimetric and volumetric energy densities of Li−S batteries.

## Results

**Fig. 1:**
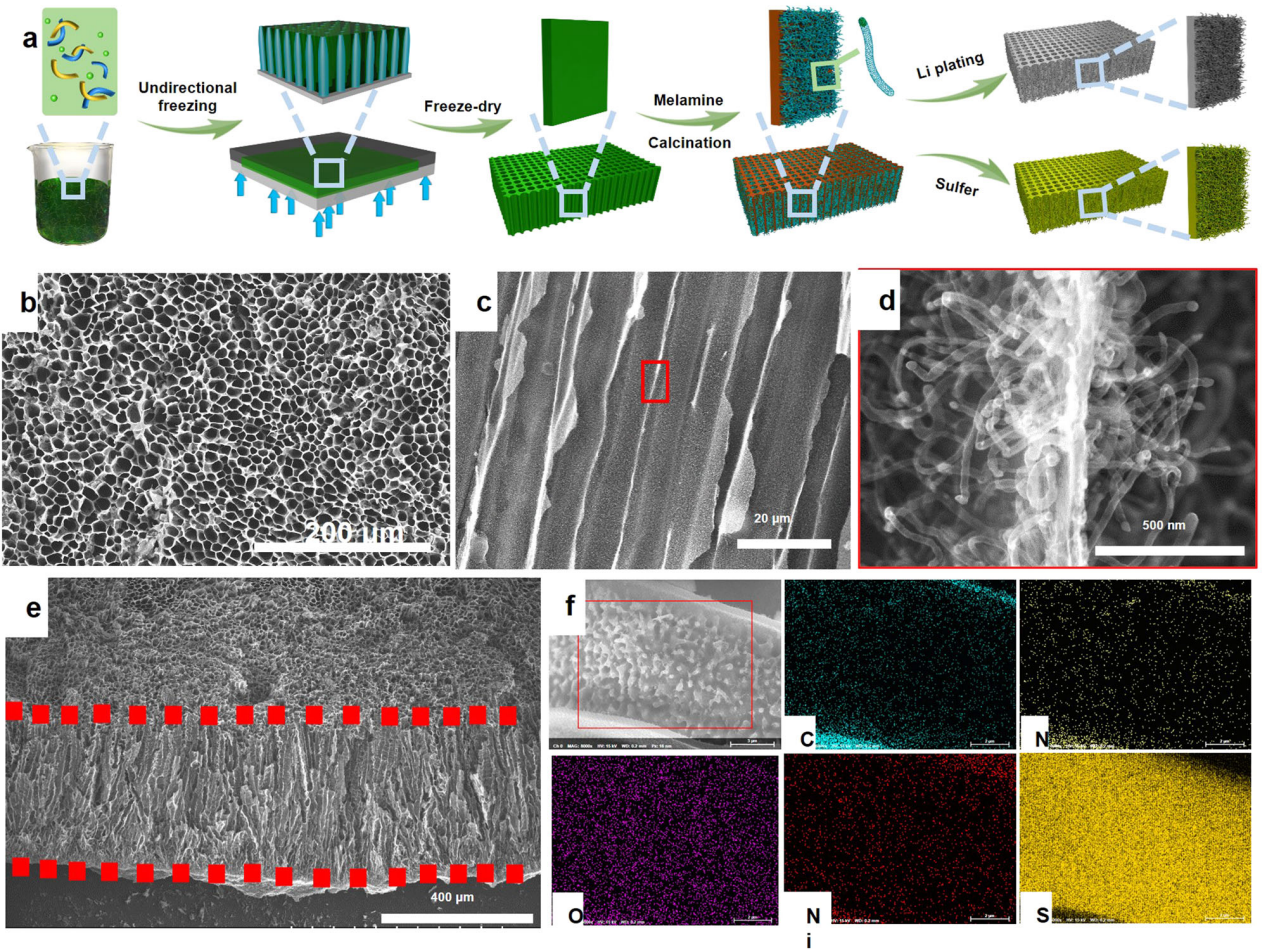
The fabrication process and design principle for S@WLC-CNTs electrodes. **a** Schematic illustration of the synthesis process, **b**−**d** Scanning electron microscope (SEM) images of the WLC-CNTs host. **e**, **f** SEM image with corresponding energy dispersive spectroscopy (EDS) maps of the S@WLC-CNTs cathode with sulfur loading of 17.3 mg cm^−2^.

Free-standing S@WLC-CNTs electrodes with low-tortuosity were fabricated via the ice-templating method and followed by a tip growth carbon nanotubes process, as illustrated in Fig. 1a. First, a small quantity of chitosan, serving as a temporary soft template during the ice sublimation process, was added to low-molecular-weight phenolic resol to form a little brown solution^19,20^. Then, a certain amount of Ni(CH_3_COO)_2_·4H_2_O, acting as a catalyst for carbon nanotubes growth, was dissolved into the brown solution, and formed a green gel-like precursor. Next, unidirectional freezing was performed to generate an ice crystals template, which grew in parallel along the *c*-axis across the whole green precursor. The ice crystals were removed by freeze-drying, presenting a wood-like architecture, which is composed of parallel channels with a diameter size of 10‒15 μm and a wall thickness around 50 nm, as well as the uniform distribution of C, N, and Ni elements, as demonstrated by scanning electron microscope (SEM) images of a vertical section and a cross-section (Supplementary Fig. S1a−h).

Furthermore, to functionalize the 3D wood-like framework, N, O co-doped carbon nanotubes (denoted as N, O-CNTs) were introduced using nickel ions (the Ni(CH_3_COO)_2_·4H_2_O added before) as the catalyst and melamine as the carbon and nitrogen source, which can generate more electron transfer paths and more specific surface area to achieve high sulfur loading and reduce the LiPS shuttle effect^21,22^. The newly formed carbon material completely inherited the wood-like architecture with aligned channels from the precursor (Fig. 1a, b). However, by magnifying the marked area in Fig. 1b, carbon nanotubes forest is observed on both sides of carbon walls with an average length of around 500 nm and a radius of around 20 nm (Fig. 1c, d). The carbon nanotubes can not only host more sulfur, but also anchor the LiPS during cycling, therefore increasing the energy density and cyclic stability of batteries. It is noticeable that nickel particles are wrapped at the top of the N, O-CNTs, and their size is consistent with the diameter of N, O-CNTs, demonstrating that they grew following the tip growth mechanism (Supplementary Fig. S2)^21,22^. In addition, the clear lattice fringes of the carbon nanotube demonstrate the high graphitization degree, indicating its high electronic conductivity. Furthermore, the carbon nanotubes forest enlarges the specific surface area of composite, which will allow more active sites for sulfur loading. As shown in Supplementary Fig. S3 and S4, the calculated specific surface area of WLC-CNTs is 130.4 m^2^ g^−1^ based on the Brunauer−Emmett−Teller method, which is much larger than that of wood-like carbon framework (WLC) without CNTs (synthesized by directly carbonization the wood-like precursor without melamine, 80.8 m^2^ g^−1^) (Supplementary Fig. S4).

Benefitting from the self-waving behavior of CNTs and the ability of the sulfur species to precipitate on their outer surfaces, it was expected that high sulfur content could be achieved in this system^15,23,24^. The sulfur content of the whole electrode could be as high as 79%, when using the WLC-CNTs matrix with a thickness of 400 μm and sulfur loading of 17.3 mg cm^−2^ (Supplementary Fig. S5). As shown in Fig. 1e and Supplementary Fig. S5, the open vertically aligned microchannels are preserved throughout the S@WLC-CNTs cathode. With its view of the detailed structure of the microchannels, Fig. 1f demonstrates that the sulfur is mainly distributed on the N, O-co-doped CNTs within the microchannels, thus occupying the void space left in the vertical microchannels and establishing direct contact between the electrolyte and the sulfur to shorten electron transfer paths, consequently, the electrolyte permeability and the ion transfer rate are improved. In addition, the Ni nanoparticles are mainly distributed in the channel walls. By combining them with the uniform distribution of N and O heteroatoms, the WLC-CNTs framework could immobilize more sulfur and trap the soluble LiPSs via chemical/physical adsorption. Hence, the wood-like framework combined with CNTs forest can construct an inner conductive network enabling fast and continuous transfer paths for electrons, ions, and electrolytes, which is expected to yield high electrochemical performance.

**Fig. 2:**
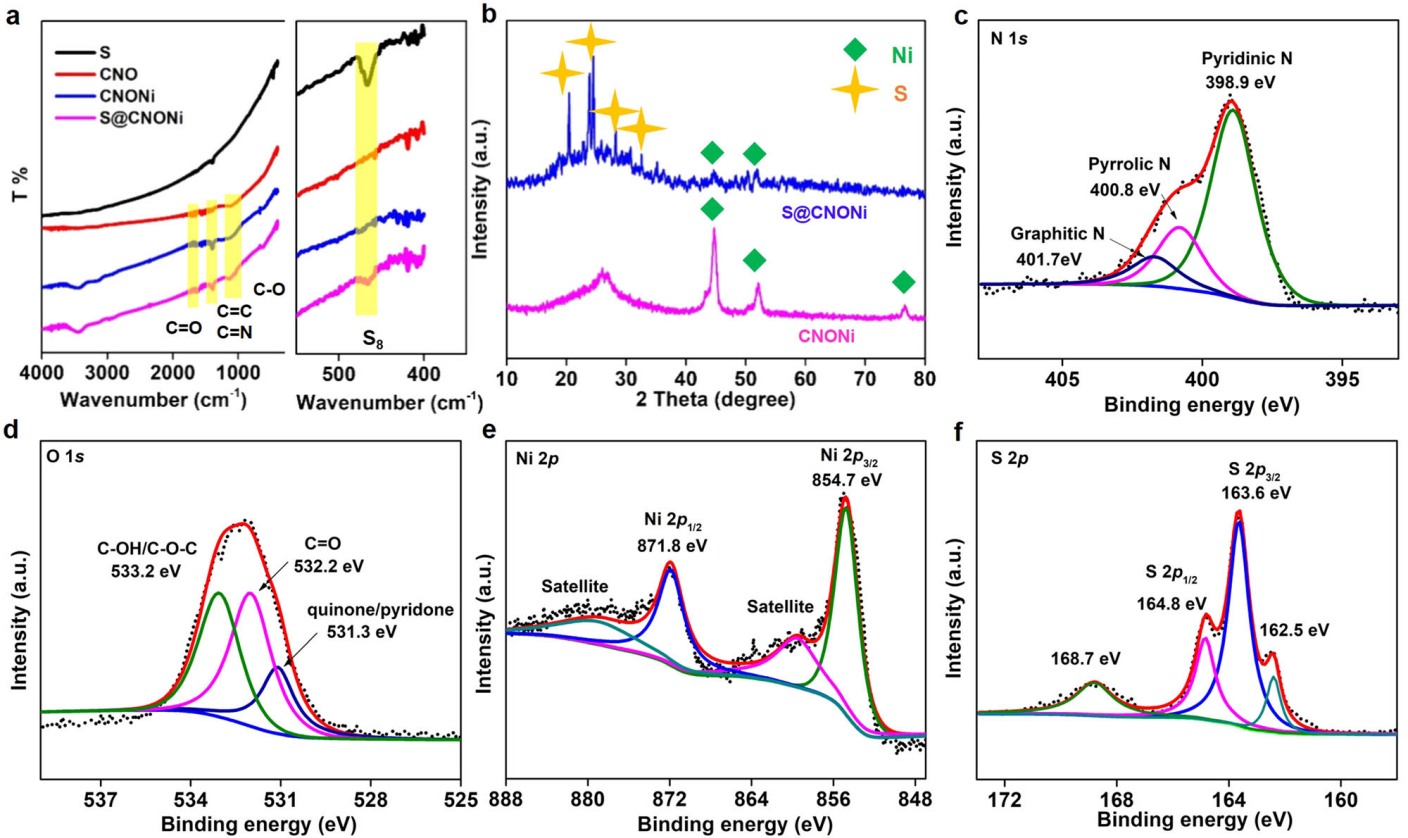
Chemical characterizations of obtained materials. **a** FTIR spectra of S, WLC, and WLC-CNTs, and S@WLC-CNTs composites. **b** XRD patterns of WLC-CNTs and S@WLC-CNTs composites. High-resolution XPS spectra of **c** N 1*s*, **d** O 1*s*, **e** Ni 2*p*, **f** S 2*p*.

The chemical composition of these samples was investigated by Fourier-transform infrared spectroscopy (FTIR), X-ray diffraction (XRD), and X-ray photoelectron spectroscopy (XPS). In the FTIR spectra (Fig. 2a), the stretching vibration modes of C=O at around 1710 cm^−1^ and C−O at 1120 cm^−1^ can be detected in the WLC-CNTs matrix. The broad peaks at 1610−1580 and 1400 cm^−1^ can be assigned to the stretching vibrations of C=C and C=N bonds, respectively. The bands at 465 cm^−1^ could be assigned to the S−S vibration mode of elemental S_8_ sulfur in the S@WLC-CNTs composite. The newly emerged weak peak at 408 cm^−1^ could be assigned to the bending and stretching modes of S−Ni^25^. The peaks related to carbon, sulfur, and nickel are detected in XRD patterns (Fig. 2b). However, the peaks related to Ni_*x*_S are not observed in XRD spectra, which is because the low-temperature heating process could not form it or because it was present at extremely low content.

Furthermore, the chemical states of the S@WLC-CNTs electrode (Supplementary Fig. S6, S7, and Fig. 2c−f) were examined by XPS. The XPS survey spectrum demonstrates the presence of C, N, O, Ni, and S elements. Three peaks located at 284.6, 285.9, and 289.0 eV in the high-resolution C 1*s* spectrum can be assigned to the C−C, C−N, and C=O bonds, respectively (Supplementary Fig. S5b). The high-resolution N 1*s* peak can be deconvoluted into three peaks located at 398.9, 400.8, and 401.7 eV, which are attributed to pyridinic N, pyrrolic N, and graphitic N (Fig. 2c). Three peaks located at 531.3, 532.2, and 533.2 eV in the high-resolution O 1*s* spectrum can be attributed to the quinone/pyridone, C=O, and C−OH/C−O−C bonds, respectively (Fig. 2d)^26,27^. The peak positions of Ni 2*p*_1/2_ and Ni 2*p*_3/2_ are higher than those of metallic Ni (Fig. 2e), which is because the overlapping of Ni 3*d* and C 2*p* states can generate covalent bonds between Ni and C, resulting in the transfer of free electrons from the interior of Ni nanoparticles to the highly N, O co-doped graphitic carbon layers^28^. The carbon surface, enriched by electrons, can facilitate the chemisorption of LiPSs. Moreover, two main peaks of S 2*p* centered at 163.6 and 164.8 eV were recognized as the S 2*p*_3/2_ and S 2*p*_1/2_ peaks of elemental sulfur. Two weak peaks at 168.7 and 162.5 eV were assigned to the S−O bonds, due to the slight oxidation, and S−Ni bonds, respectively, which is consistent with the FTIR (Fig. 2f)^26,27^. Based on XPS analysis, the concentrations of doped N and O atoms are 5.6 and 3.5 at.% in the WLC-CNTs composite. The high-level N, O, co-doping, and the nickel particles distribution can cause a shift in the Fermi level to the conduction band, generating a tunable interface for the carbon framework, promoting its reactivity, and enhancing its chemical interaction with sulfur and LiPSs, which are beneficial to Li−S battery performance^23,29^.

**Fig. 3:**
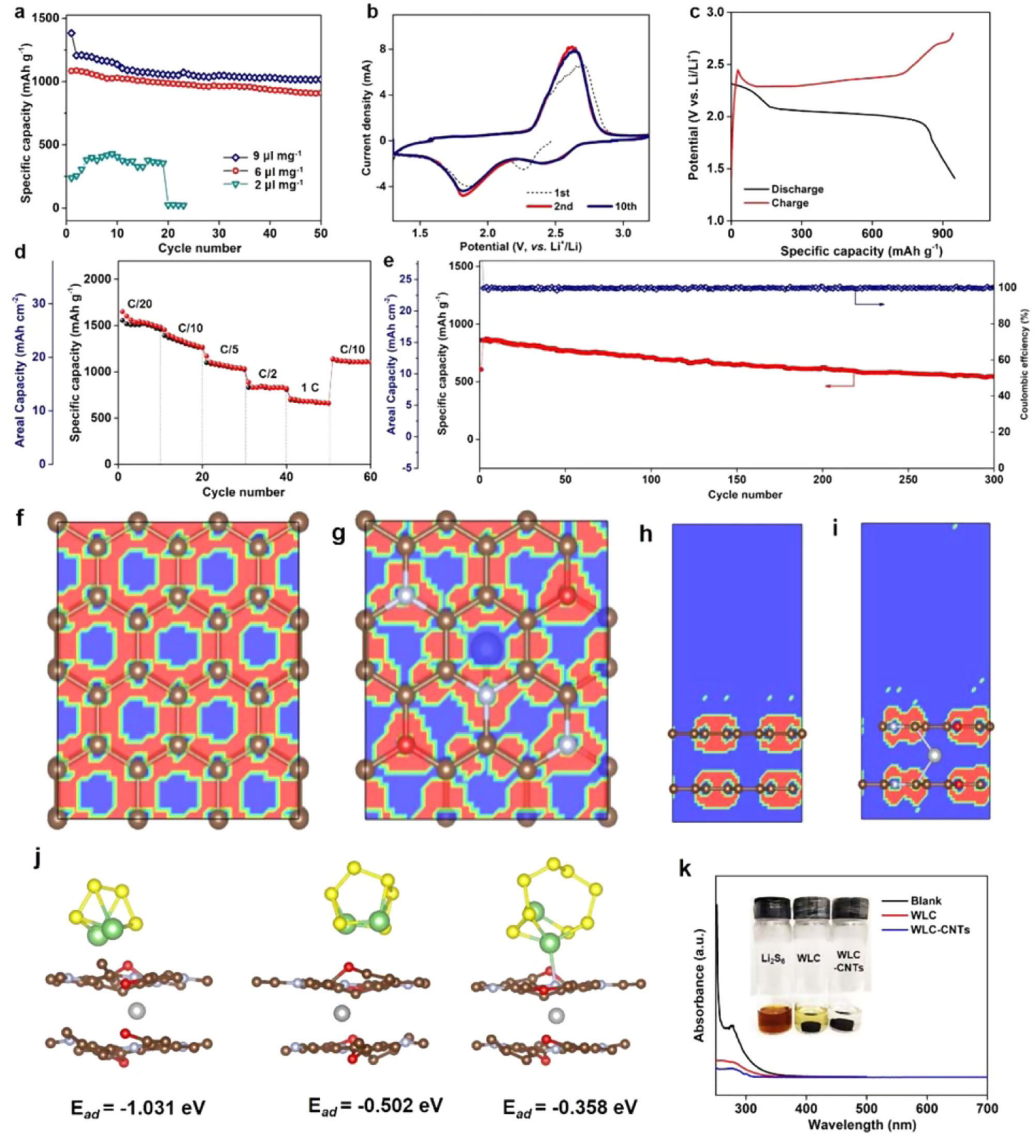
Electrochemical performance of S@WLC-CNTs with a thickness of 400 μm and sulfur loading of 17.3 mg cm^−2^. **a** Cycling performance with different amounts of electrolyte at 0.1 C (1 C = 1650 mAh g^−1^); **b** CV curves; **c** 3rd charge and discharge curves at 0.1 C; **d** rate performance; **e** long-cycling performance at 0.2 C. ELF plot of a two-dimensional cut of **f** carbon along the *ab* plane, **g** WLC-CNTs along the *ab* plane, **h** carbon along the *bc* plane, and **i** WLC-CNTs along the *bc* plane. **j** Theoretical calculation of the adsorption energies of Li_2_S_*n*_ (*n* = 4, 6, and 8) on the surface of WLC-CNTs (carbon is brown, sulfur is yellow, lithium is green, oxygen is red, nitrogen is light blue, and nickel is gray between two carbon layers). **k** Ultraviolet-visible (UV−Vis) absorption spectra of the Li_2_S_6_ solution after addition of WLC and WLC-CNTs for 1 h.

The electrochemical properties of S@WLC-CNTs cathode are evaluated by galvanostatic charge−discharge process and cyclic voltammetry (CV) examination. It is well-known that the usage of electrolyte is a critical factor for balancing the mass-specific capacity and actual energy density of Li−S batteries. A high electrolyte/sulfur (E/S) ratio is always beneficial for achieving high specific capacity and excellent stability, but at the expense of energy density^6^. As shown in Fig. 3a, when the E/S ratio is 9, the discharge capacity of S@WLC-CNTs with sulfur loading of 17.3 mg cm^−2^ could be maintained at 1192 mAh g^−1^ after 60 cycles. Reducing the E/S ratio to 6, the discharge capacity was reduced to 896 mAh g^−1^. Further lowering the E/S ratio to 2, the discharge capacity decreased to almost zero, which is due to the incomplete infiltration of electrolyte and electrolyte decomposition during cycling, as well as increased electrolyte viscosity and cell impedance^6,14,30^. Therefore, in our case, an E/S ratio of 6 in the coin cells was selected to balance high discharge capacity and energy density.

The electrochemical kinetic behavior of S@WLC-CNTs (thickness of 400 μm and sulfur loading of 17.3 mg cm^−2^) was tested by CV at a scan rate of 0.05 mV s^−1^. In the first scan, the reduction peak at 2.25 V, corresponding to the reduction from sulfur to high-order LiPSs, is at a slightly lower position than that in the following cycles, which is due to the higher kinetic barrier for the reduction of sulfur to LiPSs in initial cycle^12,31^. In the subsequent cycles, two cathodic peaks at 2.35 and 1.85 V correspond to the two-step redox reaction of sulfur to high-order LiPSs and then to the final product Li_2_S. The broad continuous anodic peak at around 2.6 V represents the oxidation reaction from Li_2_S_2_/Li_2_S to Li_2_S_8_/S^2,32^. After the first scan, there is no change in the peak positions and currents in the following cycles, demonstrating that the wood-like framework can efficiently trap the soluble LiPSs. Fig. 3c shows the discharge/charge curves, which are consistent with CV curves. There are two clear plateaus, which are typical of Li−S batteries. The higher plateau is attributed to the reduction of sulfur powder to high-order LiPSs, while the lower plateau is assigned to their further reduction to the final products of lithium sulfide.

The rate performance of S@WLC-CNTs was evaluated by discharged/charged at various current densities from C/20 to 1 C. As shown in Fig. 3d, the electrode displayed discharge capacities of 1485, 1304, 1052, and 840 mAh g^−1^ at current densities of C/20, C/10, C/5, and C/2, respectively, corresponding to 25.69, 22.56, and 18.21, and 14.54 mAh cm^−2^ at 1.42, 2.85, 5.70, and 14.27 mA cm^−2^. Even at high current density at the 1 C rate (28.54 mA cm^−2^), this electrode could still deliver about 680 mAh g^−1^ (11.77 mAh cm^−2^). Importantly, the discharge capacity could be recovered to 1105 mAh g^−1^ (19.13 mAh cm^−2^) when the current density was back to the C/10 rate, indicating the good structural stability of these electrodes at various rates (Supplementary Fig. S8).

Additionally, the long cycling performance was evaluated at 0.2 C rate. Remarkably, the capacity obtained under 0.2 C is the highest capacity among the reported testing rates for Li−S cells with low E/S ratios and high sulfur loading cathode^14^. As shown in Fig. 3e, the S@WLC-CNTs electrode delivered a discharge capacity of 862 mAh g^−1^ for the second cycle, and it still maintained a capacity of 547 mAh g^−1^ after 300 cycles, with an average capacity fading of 0.12% per cycle. Furthermore, the CE for the electrode was above 98% during the whole cycling process, illustrating that the integrated electrode can effectively trap soluble LiPSs.

The N, O co-dopants and nickel particles can make the skeleton electron-rich so that it could chemically absorb LiPSs, and the integrated electrode also physically maintains the soluble polysulfides within the electrode. This result is further confirmed by the calculation data. As shown in Fig. 3f−i, the electron localization function (ELF), where the blue region represents almost no electron density, and the red region indicates high electron density in the structure. The N and O atoms participate in the delocalized π system of carbon, thus having a strong degree of localized electron density and higher electronegativity. The Ni atom (Fig. 3i) appears to have no electron density, indicating the electrons flow onto the C atoms, making carbon skeleton electron-rich. Therefore, the adsorption ability of Li_*2*_S_*n*_ on the WLC-CNTs is better than that on the carbon surface (Fig. 3j and Supplementary Fig. S9), indicating the strong interaction between the WLC-CNTs and LiPSs. Especially for Li_2_S_4_, the adsorption energy of WLC-CNTs is −1.031 eV compared to 0.101 eV that of carbon, demonstrating a much stronger adsorption capability of Li_2_S_4_ on WLC-CNTs than that of carbon. This is consistent with the visual LiPSs adsorption experiments (Fig. 3k and Supplementary Fig. S10). A 1 mM Li_2_S_6_ solution containing WLC-CNTs becomes almost colorless after 1 h (Supplementary Fig. S10), showing significantly decreased absorption peaks in the ultraviolet-visible region (Fig. 3k)^33^. With the decoration of N, O-co-doped CNTs, the WLC-CNTs framework effectively entraps the LiPSs due to the synergistic effect of N, O-co-dopants, Ni nanoparticles, and CNTs, illustrating its stronger chemical and physical interaction with LiPSs.

**Fig. 4:**
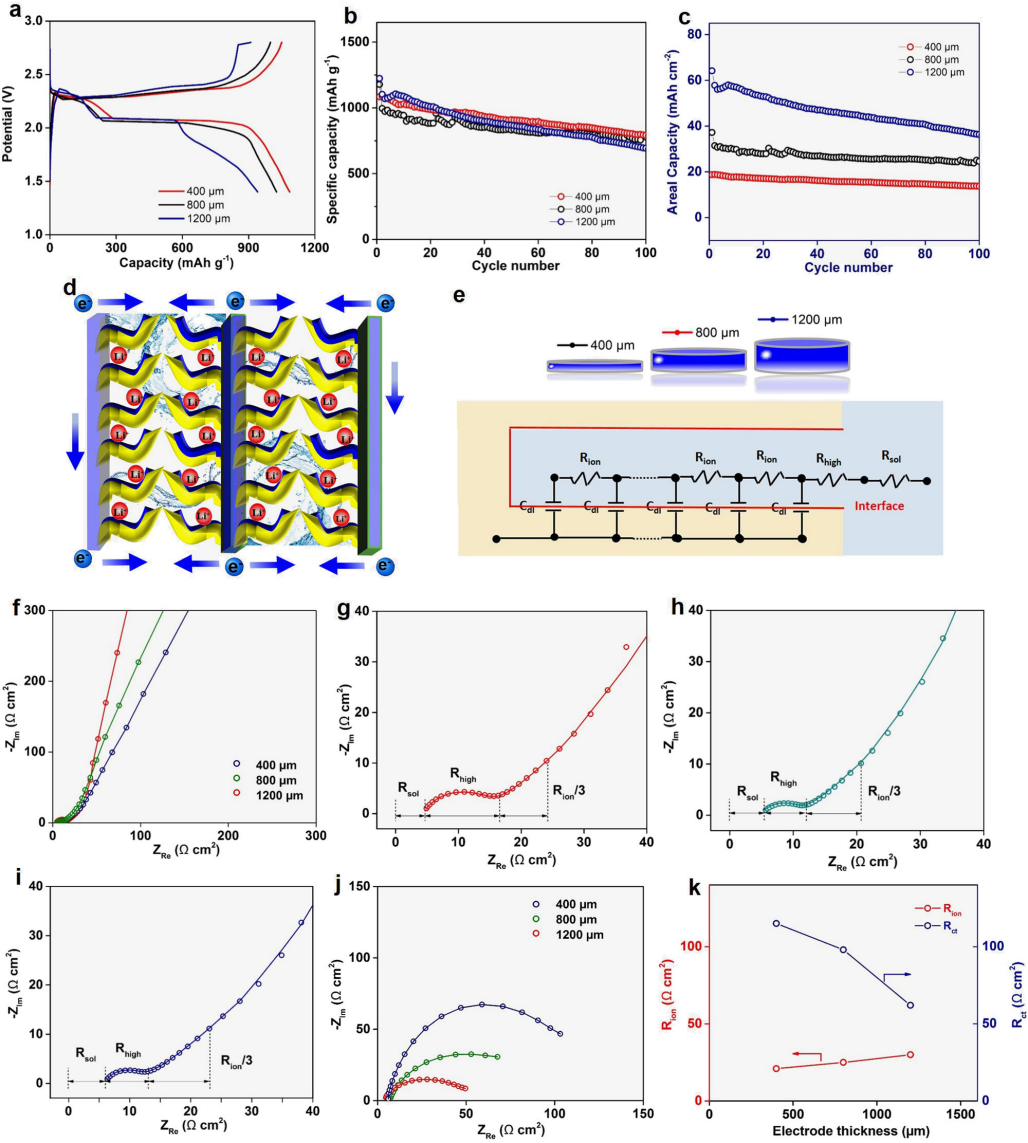
Electrochemical performance and kinetic analysis of S@WLC-CNTs electrodes with different thicknesses and areal sulfur loading. **a** Tenth cycle discharge/charge curves and **b**, **c** cyclability of S@WLC-CNTs electrodes with different thicknesses and sulfur content at the 0.1 C rate. **d** Conceptual schematic representation of Li-ion flow and electron transfer in the integrated S@WLC-CNTs electrode. **e** Equivalent circuit using the transmission line model. **f** Nyquist plots of symmetric cells with different thicknesses using two identical positive S@WLC-CNTs electrodes at 0% SOC. Magnified Nyquist plot of S@WLC-CNTs electrodes with thicknesses of **g** 400 μm, **h** 800 μm, and **i** 1200 μm. **j** Nyquist plots for symmetric cells with different thicknesses using two identical positive S@WLC-CNTs electrodes at 50% SOC. **k** *R*_ion_ and *R*_ct_ comparison between electrodes with different thicknesses.

To further explore their potential for practical application, S@WLC-CNTs electrodes with different thicknesses were fabricated. The areal sulfur loading can be as high as 31.6 mg cm^−2^ at a thickness of 800 μm and 52.4 mg cm^−2^ at a thickness of 1200 μm, which are much higher than the slurry-coated electrodes generally reported in literature^26^. As shown in Fig. 4a, even at the high areal sulfur loading of 52.4 mg cm^−2^, the discharge/charge curves still can display clear plateaus and no increased polarization, which is consistent with the electrochemical impedance spectroscopy (EIS) spectra. In the cycling performance (Fig. 4b, c), after 100 cycles, a reversible capacity of 791 mAh g^−1^ for 400 μm, 778 mAh g^−1^ for 800 μm, and 692 mAh g^−1^ for 1200 μm were obtained, corresponding to 13.69 mAh cm^−2^ at 28.54 mA cm^−2^, 24.58 mAh cm^−2^ at 52.14 mA cm^−2^, and 36.30 mAh cm^−2^ at 86.46 mA cm^−2^, respectively. The result is much higher than those of state-of-the-art LIBs (4 mAh cm^−2^)^34^.

As illustrated in Fig. 4d, the vertical microchannels in the wood-like structure are very uniform and do not change with thickness, which can improve electrolyte permeability, enabling fast reactions between Li^+^ ions and sulfur. Besides, the S@WLC-CNTs electrode can provide a highly conductive skeleton to enable homogeneous conductivity throughout the whole electrode, and the vertically aligned microchannels with low tortuosity can serve as Li^+^ ions transport highways to promote the overall electrochemical kinetics. Moreover, the carbon nanotubes forest acts as multi-layer fences to prevent the LPSs from shuttling. All these factors permit the high sulfur loading and thick electrode performance. The internal resistance of the electrode/electrolyte interface greatly affects the electrochemical capability of thick electrode. Compared to the conventional EIS tests of half/full-cells with the issue of overlapping profiles for the anode and cathode, EIS-symmetric cells (EIS-SC) using identical electrodes were adopted to decouple the internal resistance. Electrochemical processes in porous electrodes include the electrolyte bulk resistance (*R*_sol_), the ionic resistance in pores (*R*_ion_), an electric double layer at the electrode/electrolyte interface (*C*_dl_), and charge transfer resistance (*R*_ct_). All of them can be interpreted by the transmission line model theory (Fig. 4e)^16,17,35,36^. Fig. 4f shows Nyquist plots of S@WLC-CNTs electrodes with different thicknesses at 0% state of charge (SOC), which shows no faradic process, but are composed of a deformed semicircle, a straight line with a slope of 45° to the real axis, and a following vertical line. Their magnified curves are shown in Fig. 4g−i, where the electrolyte bulk resistance *R*_sol_ and the ionic/electric resistance at the interface between the electrolyte and electrode (*R*_high_) are almost the same with increasing thickness. The calculated *R*_ion_ is 21, 25, and 30 Ω cm^2^, for electrodes with a thickness of 400, 800, and 1200 μm, respectively, showing no obvious change and indicating that increasing electrode thickness has no influence on fast ionic transport.

To examine their *R*_ct_, EIS-SC for S@WLC-CNTs electrodes with different thicknesses were conducted at SOC of 50%. As shown in Fig. 4j, k, *R*_ct_ is inversely proportional to the electrode thickness, which is 120 Ω cm^2^ for 400 μm, 85 Ω cm^2^ for 800 μm, and 52 Ω cm^2^ for 1200 μm. From the viewpoint of the electrode, increasing the thickness of the electrode corresponds to varying the reaction surface area and pore length. Hence, *R*_ct,*A*_, which is defined as the charge-transfer resistance per unit electro-active surface area, can be used to fairly compare it in electrodes with various thicknesses. It is expressed as^35^

1 $${R}_{{\rm ct},A}=2\pi rL{R}_{\rm ct}$$

where *r* is the radius of the electrode and *L* is the thickness. According to our EIS-SC results that *R*_ct_ is inversely proportional to the electrode thickness, therefore, the value of *R*_ct,*A*_ for different thicknesses are almost the same. Overall, in this system, *R*_ion_ and *R*_ct,*A*_ are almost unchanged as the electrode thickness varies, which accounts well for their thickness-independent electrochemical performance.

In most reported Li−S work, a heavy Li foil anode is usually adopted, resulting in several challenges. The most critical challenge is that repeated Li plating and stripping leads to mossy and dendritic Li growth, which results in the unstable solid-electrolyte interphase (SEI) layers, Li loss during cycling, drying out of the electrolyte, and low CE due to the continuous side reactions between the liquid electrolyte and the Li metal. In addition, the overly excessive lithium will result in low energy density of Li−S batteries. Hence, to design a stable lithium metal anode at its minimum amount, it is vital to develop Li−S full batteries with high-energy density. In this work, the WLC-CNTs framework with specific lithiophilic properties was investigated as the Li host to solve the above-mentioned problems^37,38^.

**Fig. 5:**
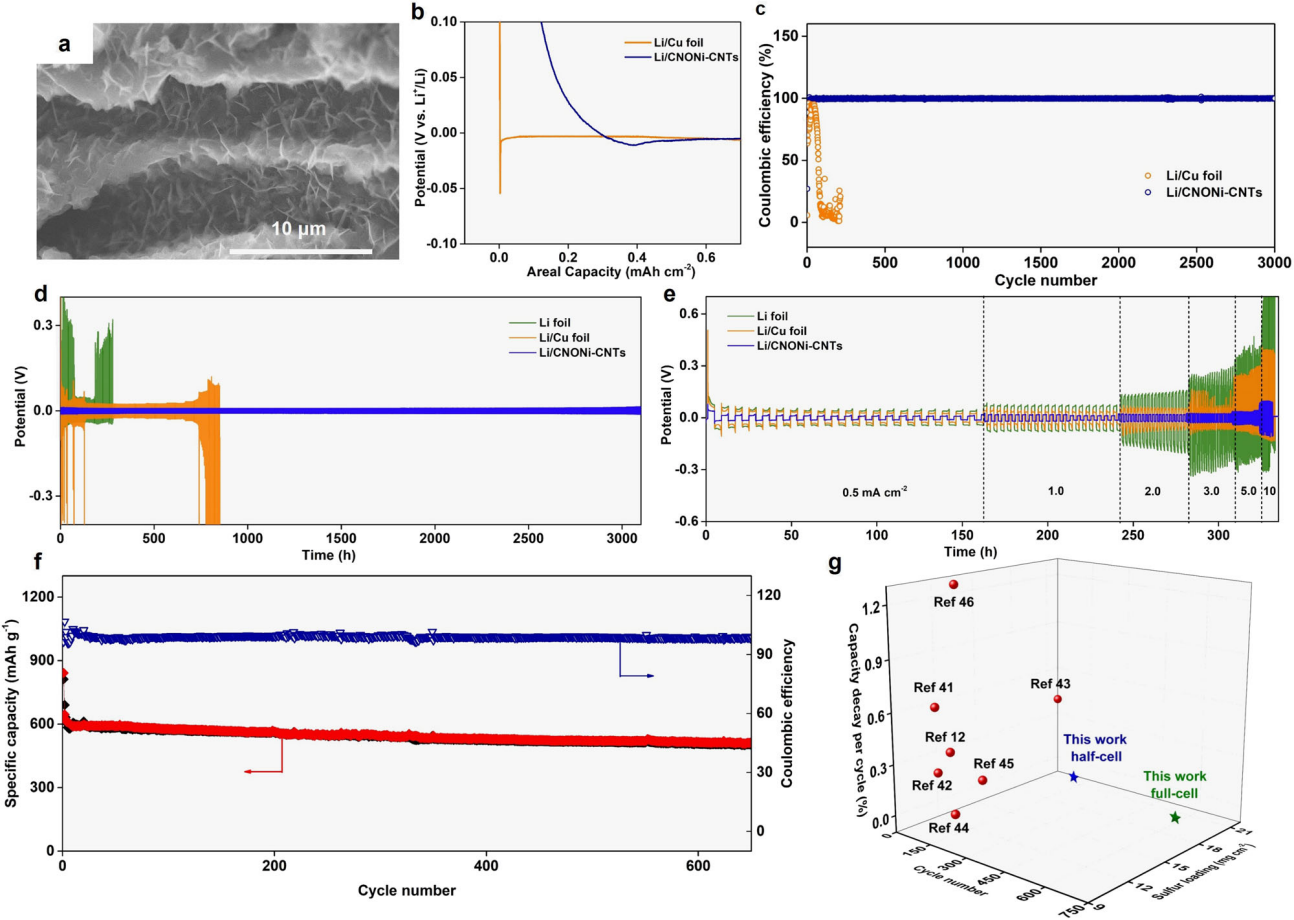
Plating/stripping behavior of lithium anode and electrochemical performance of lithium−sulfur full batteries. **a** SEM image of the 400 μm WLC-CNTs electrode with the amount of Li plating for 10 mAh cm^−2^ at 1 mA cm^−2^. **b** Nucleation behavior of Li metal on different hosts at a current density of 0.05 mA cm^−2^. **c** Comparison of the Coulombic efficiency of Li plating/stripping on the WLC-CNTs electrode and a planar Cu electrode with current density of 1 mA cm^−2^ at a fixed areal capacity of 1 mAh cm^−2^. **d** Galvanostatic plating/stripping profiles in Li foil, Li/Cu foil, and Li/WLC-CNTs symmetric cells at 1 mA cm^−2^ with areal capacity fixed at 2 mAh cm^−2^. **e** Rate performance of the symmetric cells with a fixed capacity of 2 mAh cm^−2^. **f** Cycling performance and **g** comparison of S@WLC-CNTs | |Li/WLC-CNTs full cells with other works.

Compared to the uneven lithium dendrites on planar Cu electrode (Supplementary Fig. S11), the cross-sectional SEM images of WLC-CNTs (Fig. 5a and Supplementary Fig. S12) illustrate that the electrodeposition of Li nanoflakes is quite uniform. The nucleation behavior of Li metal on Cu foil and the WLC-CNTs framework during the first electroplating process was analyzed, as shown in Fig. 5b, where the nucleation overpotential of WLC-CNTs was only 9 mV, much smaller than that of Cu (54 mV). The DFT calculation result is in agreement with these experimental results. The adsorption of a Li atom is quite sensitive to dopant elements. WLC-CNTs with N and O dopants exhibit larger binding energy (−1.711 and −4.093 eV) than that of pristine carbon (−1.068 eV) (Supplementary Fig. S13). Therefore, the introduction of N and O heteroatoms into WLC-CNTs hosts is beneficial for Li nucleation, which is consistent with experimental results that the Li nucleation overpotential has been significantly reduced and thus promotes a uniform Li deposition.

Furthermore, the CE of the ratio of the stripped Li to the plated Li capacity was examined to evaluate its reversibility, as shown in Fig. 5c. The Cu foil electrode had a low CE in the first dozen cycles and then showed serious fluctuation, which is a typical sign of the uncontrollable growth of Li dendrites^39^. In contrast, the CE of WLC-CNTs could remain above 99.8% for 3000 cycles (1 mA cm^−2^; 1 mAh cm^−2^). Besides, WLC-CNTs could also show a stable cycle life over 300 cycles at high current (3 mA cm^−2^; 3 mAh cm^−2^), as shown in Supplementary Fig. S14.

To further test the long-term stability of Li metal anode, Li/WLC-CNTs||Li/WLC-CNTs, Li/Cu||Li/Cu, and Li||Li symmetric cells were assembled. As shown in Fig. 5d, the Li/WLC-CNTs||Li/WLC-CNTs symmetric cell exhibited a remarkably small overpotential around 10 mV, which was maintained over 3100 h without fluctuation. Whereas the Li/Cu||Li/Cu, and Li||Li symmetric cells displayed large overpotentials with random fluctuations after hundreds of hours, due to the internal short-circuits caused by Li dendrite growth. Furthermore, the WLC-CNTs framework showed an outstanding ability to stabilize metallic Li when the striping/plating process was conducted at a high rate. As displayed in Fig. 5e, when the current density was increased to high rates of 5 and 10 mA cm^−2^, the Li/WLC-CNTs||Li/WLC-CNTs symmetric cell still kept the low overpotential of 35 and 85 mV, respectively. Such outstanding cycling and rate performance are ascribed to homogeneous deposition of Li, stable SEI layer, as well as easily accessible electron/ion transport pathways (Supplementary Fig. S15).

To further demonstrate the feasibility of the WLC-CNTs framework when applied as a host material for both the sulfur cathode and the lithium anode in Li−S full cells, a S@WLC-CNTs ||Li/WLC-CNTs full cell was assembled with the positive/negative ratio fixed at 2:1. As shown in Fig. 5f, it could retain a capacity of 508 mAh g^−1^ after 690 cycles with a high CE nearly to 99.8%, corresponding to a low capacity decay rate of 0.057% per cycle. Li−S full cells with such high sulfur loading have not been tested in reported work^39,40^. Moreover, the capacity retention is higher, and the cycling lifetime is longer than most Li−S half cells with comparatively high sulfur loading (Fig. 5g)^12,41–46^. Such improvement could be attributed to the eliminated lithium dendrite growth in our system.

## Discussion

In conclusion, a low-tortuosity WLC-CNTs framework has been designed and fabricated to achieve a thickness-independent sulfur cathode with high mass loading. The free-standing sulfur cathode features robust mechanical strength, high conductivity enabled by electron-rich heteroatoms, shortened ion diffusion paths, and improved chemical adsorption of LiPSs. Moreover, the vertical microchannels demonstrate thickness-independent ionic resistance and charge transfer, allowing scaling the electrode thickness without sacrificing the electrochemical performance. In addition, the WLC-CNTs with lithiophilic properties can also be used as a host for lithium to suppress dendrite growth, enabling the S@WLC-CNTs||Li/WLC-CNTs full cells to demonstrate extremely long cycling stability. This design of electrode architecture could pave a pathway to establish Li−S full cells with high energy density.

## Methods

### Preparation of carbon framework decorated with N, O co-doped carbon nanotubes (denoted as WLC-CNTs)

#### Synthesis of low-molecular-weight phenolic resol

The low-molecular-weight phenolic resol was synthesized according to a previous report^19^. NaOH aqueous solution (0.1 M), phenol (0.60 g), and formaldehyde (2.1 mL) were reacted with each other in a flask under stirring (1 h) in 70 °C oil bath. After the reaction was over, the pH of the solution was adjusted to neutral by adding HCl solution (0.1 M). Then, the water was removed by freeze-drying. Afterward, adding 20 mL ethanol to the above obtained viscous solution to separate and remove the produced NaCl. Finally, the ethanol was evaporated by placing it in the vacuum drying oven for one day at 50 °C to get the low-molecular-weight resol. The obtained dark-yellow product should be stored in the refrigerator.

#### Synthesis of WLC-CNTs framework

First, chitosan aqueous solution (2 wt%) was prepared by adding chitosan (2 g) and CH_3_COOH (2 mL) to deionized water (100 mL) under stirring. Then, the obtained resol (50 mg) was dispersed into 1.0 g of 2 wt% chitosan aqueous solution under sonication, and followed by 0.5 mL solution that contains 60 mg of Ni(CH_3_COO)_2_·4H_2_O was added through vigorous shaking. Next, the solution was poured into a round mold placed on a copper platform which is in a container of liquid nitrogen to enable it to be unidirectionally frozen. After freeze-drying, the obtained precursor was placed on the downstream side of melamine powder (1.2 g) in a tube furnace and heating it to 180 °C and keep for 2 h, and then heating it to 650 °C under a heating rate of 2 °C min^−1^ with a 4 h dwell time to yield the final product of WLC-CNTs framework.

#### Preparation of S@WLC-CNTs

First, the corresponding mass of sulfur was melted at 160 °C on a hotplate. Then, the liquid sulfur was completely absorbed by the obtained WLC-CNTs framework through simple contact because of the highly high-sulfur-philicity. The operations were all done in the glovebox. Afterward, the composite was placed in a vial and heated to 155 °C and maintained for 12 h for further impregnation to get the final product of S@WLC-CNTs.

### Characterizations

Scanning transmission electron microscopy (STEM, Hitachi S5500, and JEOL ARM-200CF), X-ray diffraction (XRD, Rigaku MiniFlex 600), thermogravimetric analysis (TGA, PerkinElmer TGA 4000), X-ray photoelectron spectroscopy (XPS, Kratos Axis Ultra DLD), and Fourier transform infrared spectroscopy (FTIR, Nicolet iS5 FT-IR spectrometer) in the spectral area of 400–4000 cm^−1^ were used to characterize the obtained materials and electrodes.

### Electrochemical measurements

CR2032 coin cells were used for the Li−S electrochemical performance tests. The cells were assembled inside an Ar-filled glovebox, using lithium metal as the anode, Celgard 2320 as the separator, and 1 M lithium bis(trifluoromethane)sulfonimide (LiTFSI) dissolved in dioxolane and dimethoxyethane (DOL/DME, volume ratio 1:1) solvent with 1 wt% LiNO_3_ added as the electrolyte.

To prepare Li/WLC-CNTs half cells, Li foil was used as the counter/reference electrode. The half cells were first activated at 0.01–1 V at 0.05 mA cm^−2^ for three cycles before the test. For symmetrical cells or full cells, WLC-CNTs, Li foil, or Cu foil were deposited with lithium using a constant current at a fixed areal capacity according to different requirements. Then, they were extracted from the half cells to assemble symmetrical cells or full cells.

Cycling and the rate performance were tested at room temperature on a Neware battery tester at room temperature (25 °C). CV and EIS measurements were carried out on a Bio-Logic potentiostat (VMP3).

### Density functional theory calculation

The Vienna Ab initio Simulation Package (VASP) was employed to perform all calculations based on the density functional theory (DFT), using the Perdew−Burke−Ernzerhof functional with the generalized gradient approximation. The projected augmented wave potentials were chosen to describe the ionic cores, and valence electrons were considered using a plane-wave basis set with a kinetic energy cutoff of 450 eV. For our structure, geometry optimizations were performed with the force convergence smaller than 0.02 eV/Å. Monkhorst−Pack k-points of 3 × 3 × 1 was applied for all the calculations. All the atoms in models are relaxed in the calculations.

## Supplementary information

Supplementary Information

## Data Availability

The authors declare that all data supporting the findings of this study are included within the paper and its Supplementary Information files. Source data are available from the corresponding author upon reasonable request.
