# Supplementary material for: Thickness-independent scalable high-performance Li-S batteries with high areal sulfur loading via electron-enriched carbon framework
Source: Nat Commun. 2021 Jul 26;12:4519. doi: 10.1038/s41467-021-24873-4 (PMC8313709; doi:10.1038/s41467-021-24873-4)
Supplement: Supplementary file 1 — Supplementary Information [file 41467_2021_24873_MOESM1_ESM.pdf]

Supplementary Materials for

Thickness-independent scalable high-performance Li-S batteries with high areal sulfur loading via electron-enriched carbon framework

Nana Wang,[ab] Xiao Zhang,[b] Zhengyu Ju,,[b] Xingwen Yu,[b] Yunxiao Wang,[a] Yi Du, [a] Zhongchao Bai,[a*] Shixue Dou, [a] Guihua Yu[b*]

[a] Institute for Superconducting and Electronic Materials, University of Wollongong，Innovation Campus, Squires Way, Wollongong, New South Wales 2500, Australia

[b] Materials Science and Engineering Program and Department of Mechanical Engineering, The University of Texas at Austin, Austin, Texas 78712, United States

S1. Morphology characterization of wood-like precursor.

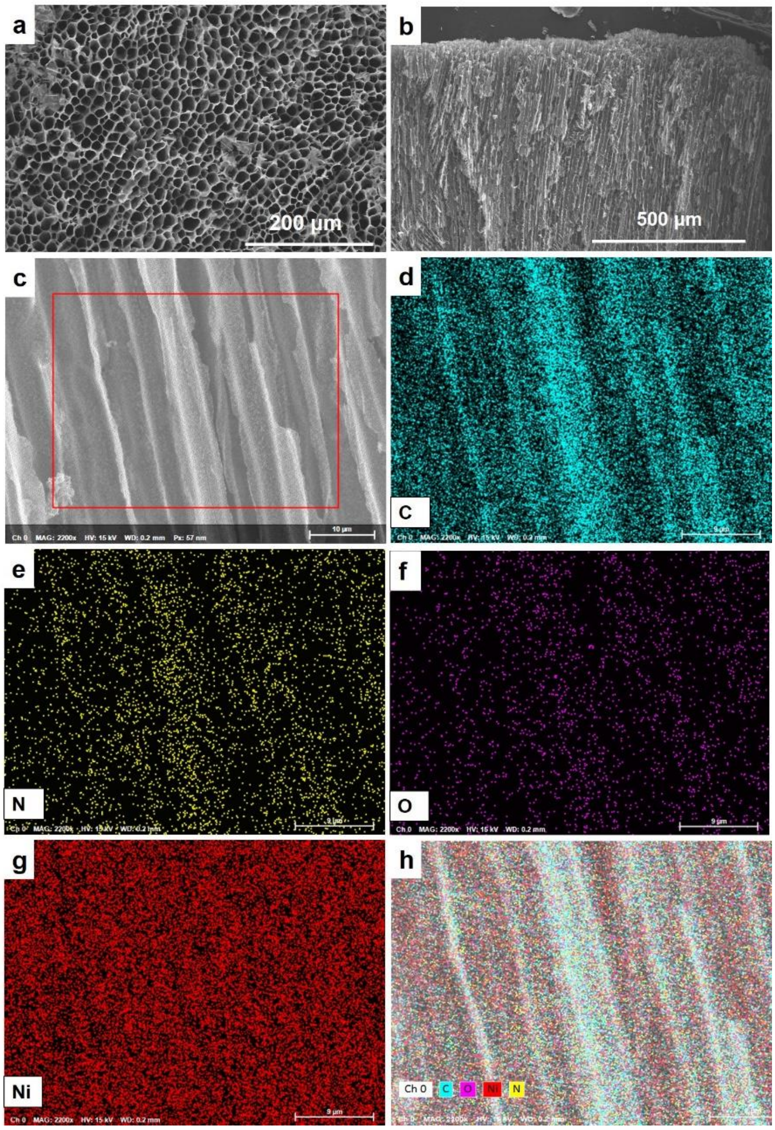

Supplementary Figure S1. Scanning electron microscope (SEM) images of (a) a cross-section and (b) a vertical section of precursor composite. (c) SEM image of precursor composite and the corresponding elemental mapping of (d) C, (e) N, (f) O, and (g) Ni.

S2. Morphology characterization of N, O-CNTs.

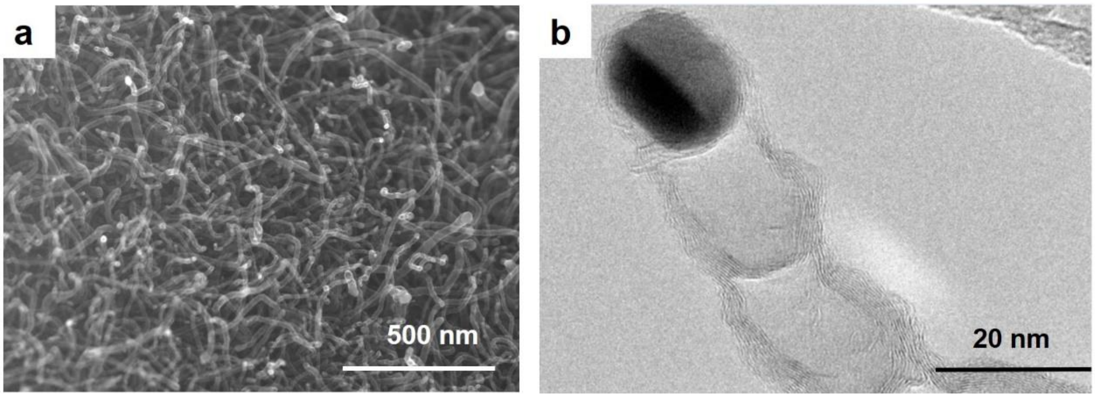

Supplementary Figure S2. (a, b) TEM images of CNONi-CNTs composite with tip growth of nickel particle.

S3. Morphology characterization of WLC.

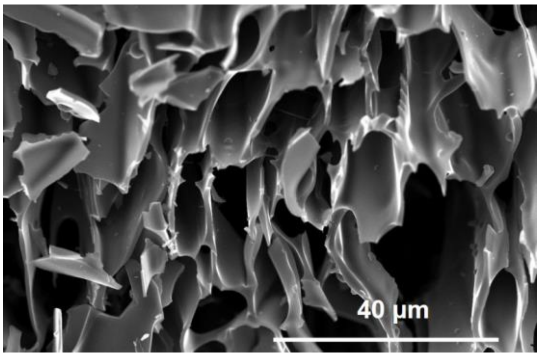

Supplementary Figure S3. SEM images of WLC.

S4. N_2 adsorption-desorption isotherms of WLC-CNTs and WLC.

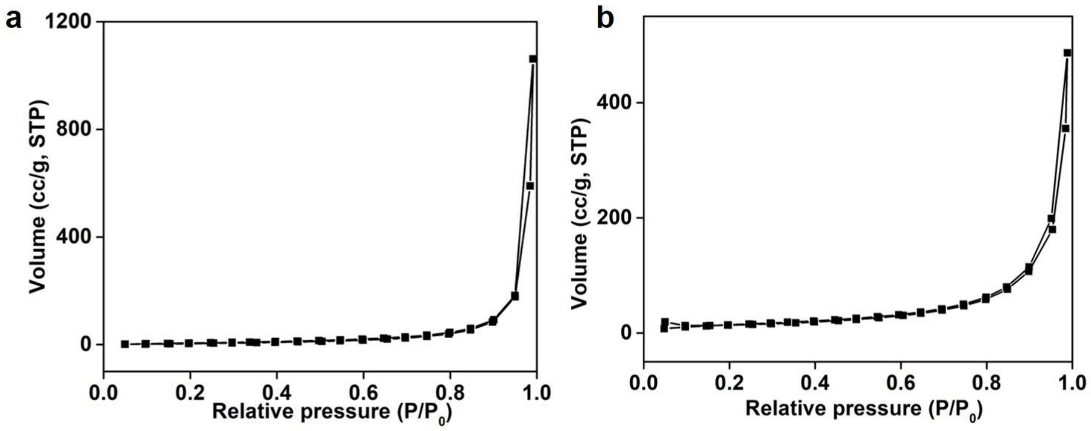

Supplementary Figure S4. N_2 adsorption-desorption isotherms of (a) WLC-CNTs composite and (b) the bare WLC carbon framework.

S5. Morphology characterization and TGA curve of S@WLC-CNTs composite.

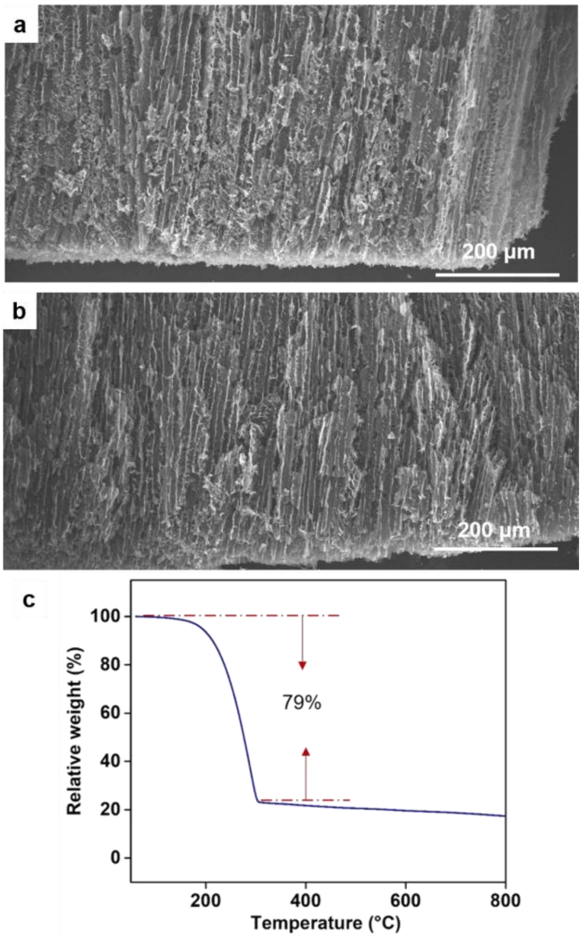

Supplementary Figure S5. (a, b) SEM images of selected different parts and (c) the TGA curve of the S@WLC-CNTs electrode with thickness of 400 μm.

S6. XPS characterization of S@WLC-CNTs composite.

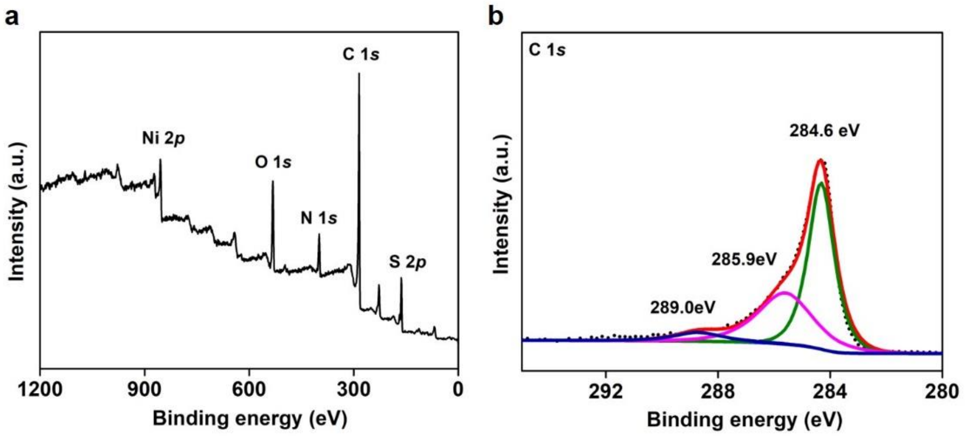

Supplementary Figure S6. (a) XPS survey spectrum of S@WLC-CNTs composite. (b) High-resolution XPS spectrum of C 1*s*.

S7. XPS characterization of WLC-CNTs composite.

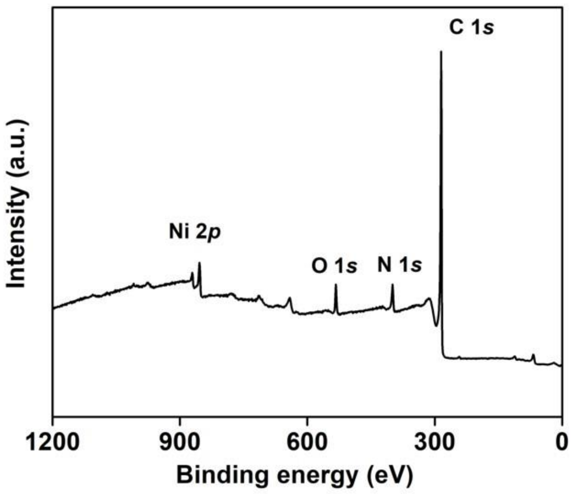

Supplementary Figure S7. XPS survey spectrum of WLC-CNTs composite.

S8. Post-cycling of S@WLC-CNTs composite.

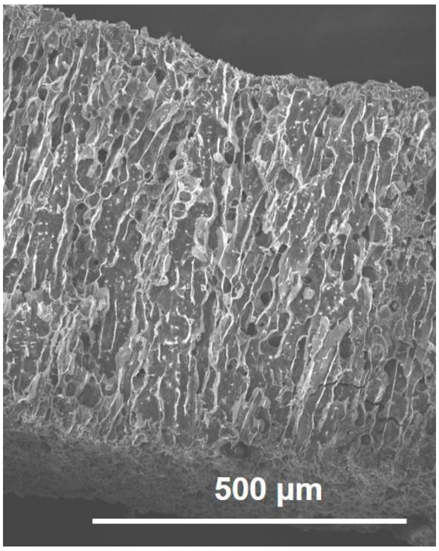

Supplementary Figure S8. SEM images of S@WLC-CNTs composite after rate cycling.

S9. Adsorption energies of Li_2S_n (n = 4, 6, 8) on the surface of carbon.

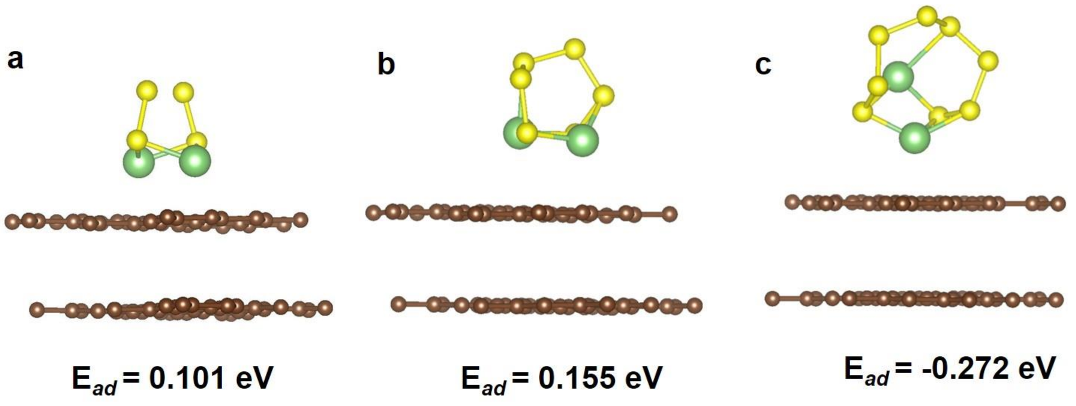

Supplementary Figure S9. Theoretical calculation of the adsorption energies of Li_2S_n (n = 4, 6, 8) on the surface of carbon.

S10. Visual illustration of polysulfide entrapment.

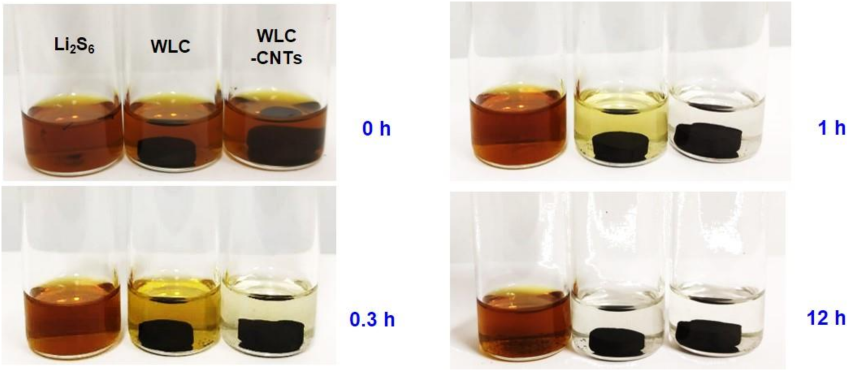

Supplementary Figure S10. Digital images of the sealed vials with Li_2S_6 solution in DOL and DME during contact in the WLC, WLC-CNTs adsorption test.

S11. SEM images of Li/Cu foil electrode.

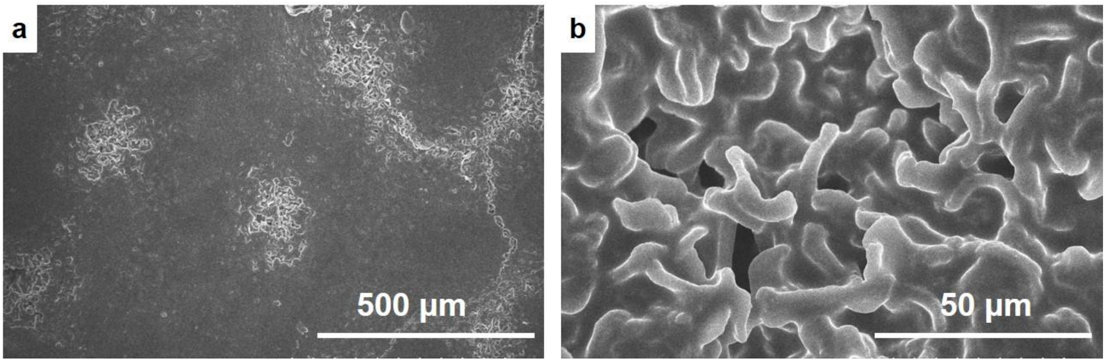

Supplementary Figure S11. SEM images of the planar Cu electrode with the amount of Li plating for areal capacity of 10 mAh cm^{-2} at 1 mA cm^{-2}.

S12. SEM images of Li/WLC-CNTs electrode.

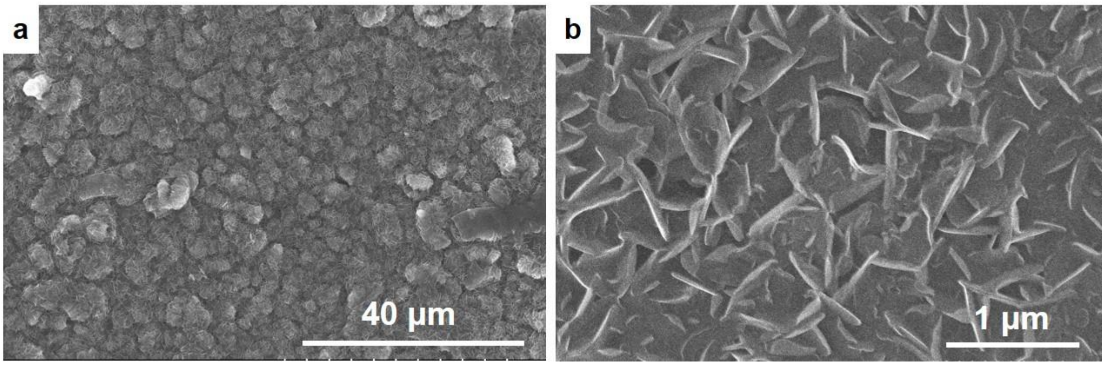

Supplementary Figure S12. SEM images of the top of the 400 μm WLC-CNTs electrode with the amount of Li plating for areal capacity of 10 mAh cm^{-2} at 1 mA cm^{-2}.

S13. Modeling of Li binding energy.

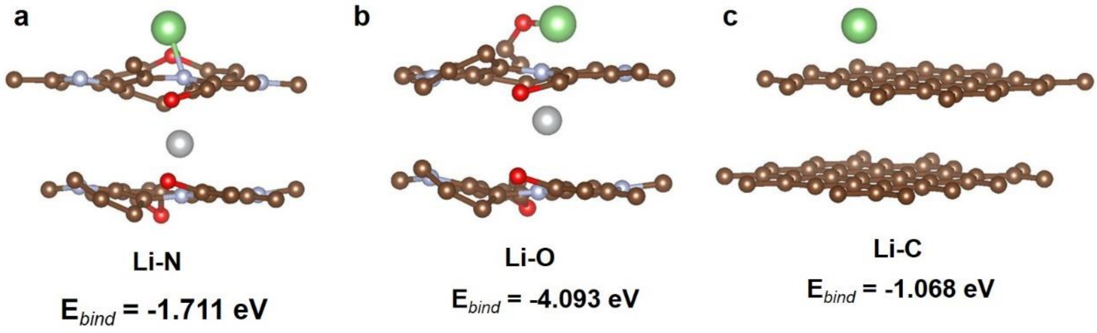

Supplementary Figure S13. Modeling of Li binding energy with (a, b) WLC-CNTs and (c) carbon surface (carbon is brown, lithium is green, oxygen is red, nitrogen is light blue, and nickel is grey between two carbon layers).

S14. The Coulombic efficiency of Li/WLC-CNTs and Li/Cu electrodes.

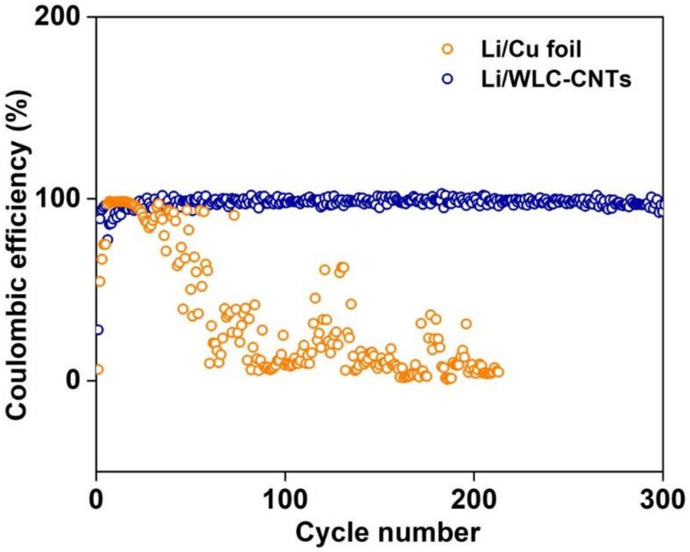

Supplementary Figure S14. Comparison of the Coulombic efficiency of Li plating/stripping on the WLC-CNTs electrode and the planar Cu electrode with current density of 3 mA cm^{-2} at fixed areal capacity of 3.5 mAh cm^{-2}.

S15. Post-cycling of Li/WLC-CNTs composite.

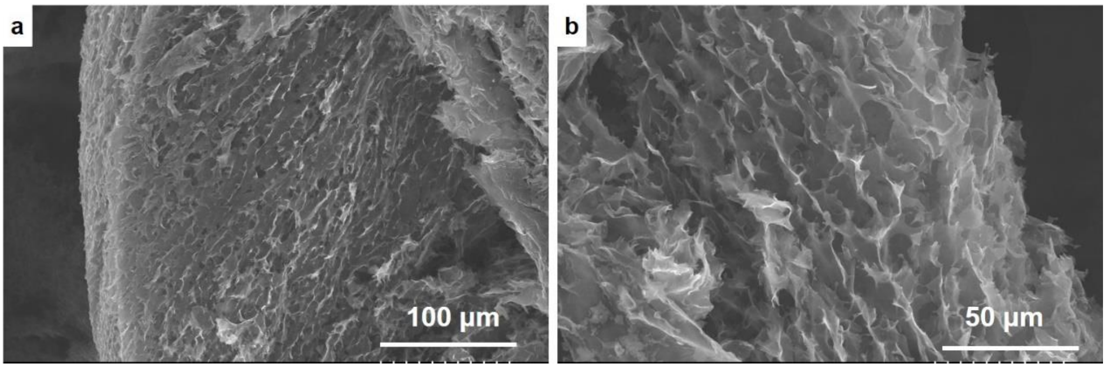

Supplementary Figure S15. SEM images of Li/WLC-CNTs composite after rate cycling.
